# A Parameter-Fitted PC-SAFT Framework for Solubility Extrapolation in Drug–Polymer Systems

**DOI:** 10.1021/acs.molpharmaceut.5c00939

**Published:** 2025-09-26

**Authors:** Alex Mathers, Michal Fulem

**Affiliations:** Department of Physical Chemistry, 52735 University of Chemistry and Technology, Prague, Technická 5, 166 28 Prague 6, Czech Republic

**Keywords:** amorphous solid dispersion, drug solubility, PC-SAFT, phase diagram, poly(2-oxazoline)

## Abstract

Accurate modeling of drug–polymer solubility is essential for the rational design of amorphous solid dispersions and other advanced pharmaceutical formulations. The perturbed-chain statistical associating fluid theory (PC-SAFT) equation of state has emerged as a robust framework for capturing complex thermodynamic interactions in such systems. However, its predictive accuracy is often constrained by the limited availability of validated pure-component parameters and the frequent need to optimize the binary interaction parameter (*k*_ij_) to match experimental data. In this study, we present a novel application of PC-SAFT as a data-driven extrapolation tool in which model parameters are directly regressed to experimental solubility data for specific drug–polymer pairs. This approach repositions PC-SAFT from a purely predictive model to a pragmatic extrapolative framework, enabling solubility estimation without reliance on pretabulated parameters or speculative *k*_ij_ adjustments. In a separate analysis, we further demonstrate that using arbitrary pure-component parameter values—when coupled with *k*_ij_ optimization—can achieve predictive performance comparable to that of literature-derived parameters. This finding underscores the dominant role of the binary interaction parameter and suggests that detailed pure-component calibration may not be essential for capturing the solubility behavior. Case studies confirm that both strategies reliably reproduce experimental trends and offer practical paths for bridging data gaps in the thermodynamic modeling of drug–polymer systems.

## Graphical abstract

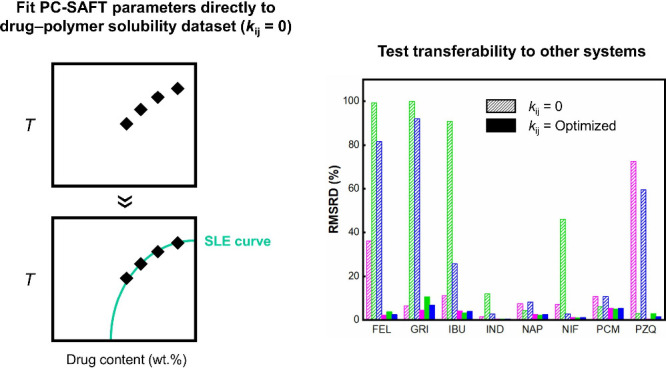

## 1 Introduction

Amorphous solid dispersions (ASDs) are a widely adopted formulation strategy to enhance the bioavailability of poorly water-soluble active pharmaceutical ingredients (APIs). Central to their success is the selection of a polymeric carrier that exhibits sufficient compatibility with the API to inhibit recrystallization and promote solubilization. Traditionally, this compatibility has been assessed through empirical screening—an approach that is both time-intensive and experimentally demanding. In response, molecular thermodynamic models such as the perturbed-chain statistical associating fluid theory (PC-SAFT) equation of state have garnered increasing attention as tools for rational formulation design.

PC-SAFT, introduced by Gross and Sadowski^1^ in 2001, models molecules as chains of spherical segments and accounts for dispersive and associative interactions via a Helmholtz energy perturbation framework. Originally developed for nonassociating fluids, PC-SAFT was quickly extended to include hydrogen bonding, polar interactions, and polymeric chain behavior, leading to its successful application to a broad range of fluids—including gases, hydrocarbons, associating solvents, and polymers.^2,3^ Compared to simpler cubic equations of state, PC-SAFT offers a more detailed molecular representation, making it well-suited for capturing the complex phase behavior of multicomponent mixtures.^4^

More recently, PC-SAFT has found use in pharmaceutical applications, particularly for predicting API–polymer compatibility in ASDs.^5^ Several studies have applied PC-SAFT to model solid–liquid equilibrium (SLE) in API–polymer systems, often showing that the model can qualitatively rank polymer performance or reproduce full temperature–composition phase diagrams.^6–10^ However, quantitative predictions frequently depend on empirical adjustment of the binary interaction parameter, *k*_ij_, to align the model output with experimental solubility data. In practice, “pure predictions” assuming *k*_ij_ = 0 tend to show poor agreement—especially in systems involving polar or associating compounds—leading many researchers to omit these baseline results or present only optimized fits.

This reliance on tuning raises fundamental questions about the necessity of obtaining accurate pure-component parameters in the first place. The standard approach to PC-SAFT parametrization involves regression to experimental vapor pressure and liquid density data, which may be unavailable or impractical to obtain for novel APIs or excipient polymers.^4^ Even when such data exist, the resulting parameters may not be transferable to API–polymer mixtures under conditions relevant to the ASD formulation. Frequently, PC-SAFT parameters for APIs are derived using solubility data in small-molecule solvents like ethanol or toluene—systems that differ substantially from the high-molecular-weight, amorphous polymers used in pharmaceutical formulations. As a result, the utility of these parameter sets for predicting API–polymer compatibility is often unclear and may depend more on downstream tuning (e.g., of *k*_ij_) than on the quality of the original parametrization.

To address these challenges, one approach explored in this study involves directly fitting the PC-SAFT parameters of the API and polymer to experimental solubility data while holding *k*_ij_ = 0 fixed throughout the fitting process. A custom Python-based program was developed to enable automated screening and optimization of candidate parameter sets based on the root-mean-square relative deviation (RMSRD) value from experimentally measured solubility curves. This method aims to evaluate whether empirical fitting to solubility data alone can yield effective parameter sets that serve as practical starting points for subsequent extrapolation or *k*_ij_ tuning—without relying on thermophysical property measurements such as vapor pressure or liquid density.

By analyzing multiple parametrization strategies across a series of well-characterized API–polymer systems, this study repositions PC-SAFT as a tool for solubility-driven extrapolation rather than strict thermophysical prediction. In doing so, it seeks to offer a practical and scalable framework for early stage formulation development, particularly in contexts where conventional parametrization pathways are infeasible.

## 2 Computational Methods

### 2.1 SLE Curve Modeling

The equilibrium solubility of an active pharmaceutical ingredient (API) in an amorphous polymeric matrix is governed by the condition of equality between the chemical potentials of the crystalline and the dissolved states. Under this condition, the mole fraction solubility of the API (*x* ^L^_API_) in the polymer can be described by the following expression:

1 $$x_{\mathrm{API}}^{L}=\frac{1}{\gamma_{\mathrm{API}}^{L}}\exp\left[-\frac{\Delta_{\mathrm{fus}}H}{RT}\left(1-\frac{T}{T_{m,\mathrm{onset}}}\right)-\frac{1}{RT}\int_{T_{m,\mathrm{onset}}}^{T}\Delta_{\mathrm{fus}}C_{p}dT+\frac{1}{R}\int_{T_{m,\mathrm{onset}}}^{T}\frac{\Delta_{\mathrm{fus}}C_{p}}{T}dT\right]$$

In eq 1, γ^L^_API_ is the activity coefficient of the API in the liquid API–polymer mixture, Δ_fus_ *H* is the pure API molar enthalpy of fusion, *T*_m, onset_ is the pure API onset temperature of melting, Δ_fus_ *C*_*p*_ is the difference in isobaric heat capacity between the supercooled liquid and crystalline API, *R* is the universal gas constant, and *T* is the temperature of interest. In practice, the term Δ_fus_ *C*_*p*_ is often poorly characterized due to the scarcity of reliable *C*_*p*_ data for the supercooled liquid phase. As such, it is common to adopt a constant Δ_fus_ *C*_*p*_ value estimated at the melting temperature *T*_m_. When this term is excluded or held constant, an accurate estimation of the activity coefficient γ_API_^L^ becomes even more critical for solubility modeling.

#### 2.1.1 PC-SAFT Overview

To calculate γ^L^_API_, this study employed the perturbed-chain statistical associating fluid theory (PC-SAFT) equation of state. This model is based on the concept of associating fluids, characterized by specific interactions between molecules leading to cluster formation. To calculate the residual Helmholtz energy (*a* ^res^), a sum of several contributions is used, taking into account the properties of individual molecules:

2 $$a^{\mathrm{res}}=a^{\mathrm{hc}}+a^{\mathrm{disp}}+a^{\mathrm{assoc}}$$

In eq 2, PC-SAFT considers repulsive forces through the term *a* ^hc^, which depends on the size and shape of molecules (the number of spherical segments *m*_i_ and their diameter σ_i_). Dispersive interactions are described by the term *a* ^disp^, which uses the dispersion energy parameter *u/k*_B_ (*k*_B_ is Boltzmann’s constant). For associating substances, it is also necessary to consider the association energy (ϵ^assoc^/*k*_B_), the association volume (κ^assoc^), and the number of hydrogen bond donors and acceptors (*N* ^assoc^) through the term *a* ^assoc^. These parameters (*m*, σ, *u/k*_B_, ϵ^assoc^/*k*_B_, κ^assoc^, and *N* ^assoc^) form the basic input for describing the substance.

In practice, a binary interaction parameter *k*_ij_ is typically employed in PC-SAFT to correct results based on experimental data. However, regarding the two-step optimization strategy applied in this study (see below), all calculations were performed with *k*_ij_ = 0. To calculate the activity coefficient of component i in a mixture, eq 3 is used:

3 $$\ln\gamma_{i}^{L}=\ln\varphi_{i}^{L}-\ln\varphi_{0,i}^{L}$$

where the activity coefficient is expressed using the fugacity coefficients of component i in the liquid mixture (φ_i_^L^) and the fugacity coefficient of pure liquid component i (φ_0,i_^L^).

The following section details the two-step parameter optimization framework developed to calibrate the PC-SAFT model against experimental solubility data. Various PC-SAFT parametrization strategies were compared in this study, which are described in more detail in the Results and Discussion section. One of these approaches involved using previously published PC-SAFT parameters for both the API and polymer components. The literature values used in this work are summarized in Table 1.

**Table 1:** PC-SAFT Parameters for the Studied Compounds Taken from the Literature

**Compound** ^a^	***M***_**w**_ ^b^ / g mol^–1^	***m*/*M*_w_** / mol g^–1^	**σ** / Å	***u*/*k*_B_** / K	***ε*** ^**assoc**^/***k***_**B**_ / K	**κ** ^**assoc**^	***N*** ^**assoc**^ ^h^
*APIs*							
FEL^c^	384.253	0.03000	3.2050	234.5000	1581.1000	0.02	4 (2, 2)
GRI^d^	352.767	0.04018	3.3720	221.3000	1985.5000	0.02	4 (2, 2)
IBU^e^	206.285	0.02637	4.0179	309.4000	516.4691	0.089457	4 (2, 2)
IND^e^	357.790	0.02207	3.8225	374.5100	1295.4320	0.011350	6 (3, 3)
NAP^e^	230.263	0.01916	4.1142	470.9200	1202.6520	0.009524	4 (2, 2)
NIF^f^	346.339	0.02347	3.5810	309.4400	1221.5800	0.02	4 (2, 2)
PCM^e^	151.165	0.02141	3.9819	432.0900	1635.9150	0.054320	4 (2, 2)
PZQ^g^	312.413	0.01991	4.0900	327.1000	0	0.02	2 (0, 2)
*polymers*							
PVP K12	2,500	0.04070	2.7100	205.599	0	0.02	44 (22, 22)
PVP VA 64	65,000	0.03720	2.9470	205.271	0	0.02	1,306 (653, 653)
SOL	118,000	0.05400	2.8090	225.000	0	0.02	4,972 (2,486, 2,486)

a FEL = felodipine; GRI = griseofulvin; IBU = ibuprofen; IND = indomethacin; NAP = naproxen; NIF = nifedipine; PCM = paracetamol; PZQ = praziquantel; PVP K12 = poly­(vinylpyrrolidone) K12; PVP VA 64 = poly­(vinylpyrrolidone-vinylacetate) 64; SOL = Soluplus.

b *M*_w_ = molecular weight.

c Taken from Luebbert et al.^8^

d Taken from Paus et al.^11^

e Taken from Klajmon.^12^

f Taken from Luebbert and Sadowski.^13^

g Taken from Brinkmann.^14^

h The parameter is presented in the format Z (X, Y), where Z is the total number of association sites, X is the number of electron donors, and Y is the number of electron acceptors.

### 2.2 PC-SAFT Parameter Optimization Framework

To enable extrapolation of experimental API–polymer solubility data, a two-step parameter optimization workflow was developed to fit PC-SAFT parameters directly to solid–liquid equilibrium (SLE) data. The goal was to minimize the root-mean-square relative deviation (RMSRD) between PC-SAFT-predicted and experimentally determined solubility values expressed as weight fractions. All calculations were performed using a custom Python 3.10-based pipeline that interfaced with a proprietary PC-SAFT executable (PC_SAFT_ASD_v2022.12.exe), which simulated the solubility behavior of binary amorphous systems.

#### 2.2.1 Parameter Definitions and Fitting Strategy

PC-SAFT requires the specification of molecular parameters for each component, including *m*, σ, *u*/*k*_B_, and, where applicable, *ε* ^assoc^/*k*_B_ and κ^assoc^. In our code, eight adjustable parameters per system, labeled A through H, were optimized for their respective experimental data set. Five parameters were fitted to the API component: the mass-normalized segment number (*m*/*M*_w_) [A], σ [B], *u*/*k*_B_ [C], and the two association parameters (*ε* ^assoc^/*k*_B_ [D] and κ^assoc^ [E]). For the polymer component, three parameters were optimized: *m*/*M*_w_ [F], σ [G], and *u*/*k*_B_ [H]. The polymer association parameters were held constant at *ε* ^assoc^/*k*_B_ = 0 and κ^assoc^ = 0.02, in accordance with typical values reported in the literature for nonassociating or weakly associating polymers.

#### 2.2.2 Step 1: Randomized Screening and Local Refinement

In the initial screening step, 20 random PC-SAFT parameter sets were generated within bounds informed by values reported in the literature (see Table S1a and Table S1b in the Supporting Information). For each set, an input file (.inp) was automatically constructed using a parametrized PC-SAFT input template, and the PC-SAFT executable was called to simulate the binary API–polymer solid–liquid equilibrium (SLE) behavior. The predicted API solubility, expressed as a weight fraction, was then compared to the corresponding experimental data, and the root-mean-square relative deviation (RMSRD) was computed for each trial. The parameter set that produced the lowest RMSRD values was identified and retained for further optimization. This best-performing set served as the initial guess for a local refinement step, which employed the Nelder–Mead simplex algorithm, as implemented in the scipy.optimize.minimize function. To maintain computational efficiency during this preliminary pass, the number of optimization iterations was limited to five.

#### 2.2.3 Step 2: Extended Local Optimization

Although the second step was originally intended as a fallback step for poorly converged fits, in this study, it was applied to all API–polymer systems to ensure methodological consistency. Starting from the best set obtained in the first step, the parameter set was subjected to 50 additional Nelder–Mead iterations to further reduce the RMSRD. This allowed for a more thorough exploration of the parameter space and improved fitting performance.

#### 2.2.4 Objective Function and Execution Environment

The objective function minimized during both optimization steps was the RMSRD, defined using weight fraction solubility (*w*_i_) as

4 $$\mathrm{RMSRD}=\sqrt{\frac{1}{N}\overset{N}{\underset{i=1}{\sum }}{\left(\frac{w_{i}^{\exp}-w_{i}^{\mathrm{calc}}}{w_{i}^{\exp}}\times 100\right)}^{2}}$$

In eq 4, *N* is the number of experimental data points, and *w*_*i*_^exp^ and *w*_*i*_^calc^ are the experimental and calculated solubilities, respectively. Optimization routines incorporate penalty terms to enforce parameter constraints and ensure convergence within physically meaningful bounds. All computations, file generation, and simulation runs were performed in a Windows environment with full automation. Intermediate and final RMSRD values were recorded, and the optimized PC-SAFT parameter sets were used for subsequent modeling of the SLE curve.

The Python scripts used for parameter sampling, input file generation, and RMSRD-based optimization are available at: https://github.com/dr-alexmathers/pc-saft-parameter-optimization. These scripts are intended to interface with a compiled PC-SAFT executable that is not publicly available. As such, the provided code is designed to demonstrate the workflow and can be adapted for use with other PC-SAFT implementations, provided that they support the required input and output file formats.

### 2.3 Experimental Data Sources for Benchmarking

All experimental data used for PC-SAFT-based solubility modeling and validation were obtained from previous studies conducted by our research group, and these are outlined in Table 2. No new experimental measurements were performed in this work.

**Table 2:** Sources of the DSC-Based API–Polymer Experimental Solubility Data Used in This Work^a^

API–polymer	Reference (Year)	API–polymer^b^	Reference (Year)
IND–PVP K12	Mathers et al.[Bibr ref15] (2021)	IBU–PEtOx-5	Mathers et al.[Bibr ref16] (2025)
NAP–PVP K12	Mathers et al.[Bibr ref17] (2023)	IBU–PEtOx-50	Mathers et al.[Bibr ref16] (2025)
NAP–PVP VA 64	Mathers et al.[Bibr ref17] (2023)	IBU–PEtOx-500	Mathers et al.[Bibr ref16] (2025)
NAP–SOL	Mathers et al.[Bibr ref17] (2023)	IND–PEtOx-5	Mathers et al.[Bibr ref16] (2025)
NIF–PVP K12	Mathers et al.[Bibr ref17] (2023)	IND–PEtOx-50	Mathers et al.[Bibr ref16] (2025)
NIF–PVP VA 64	Mathers et al.[Bibr ref17] (2023)	IND–PEtOx-500	Mathers et al.[Bibr ref16] (2025)
NIF–SOL	Mathers et al.[Bibr ref17] (2023)	NAP–PEtOx-5	Mathers et al.[Bibr ref16] (2025)
GRI–PVP K12	Mathers et al.[Bibr ref17] (2023)	NAP–PEtOx-50	Mathers et al.[Bibr ref16] (2025)
FEL–PVP K12	Mathers et al.[Bibr ref18] (2024)	NAP–PEtOx-500	Mathers et al.[Bibr ref16] (2025)
IBU–PVP K12	Mathers et al.[Bibr ref18] (2024)	PCM–PEtOx-5	Mathers et al.[Bibr ref16] (2025)
PCM–PVP K12	Mathers et al.[Bibr ref18] (2024)	PCM–PEtOx-50	Mathers et al.[Bibr ref16] (2025)
PZQ–PVP K12	Mathers et al.[Bibr ref18] (2024)	PCM–PEtOx-500	Mathers et al.[Bibr ref16] (2025)

a The stepwise dissolution method was employed to obtain the experimental solubility data for all API–polymer binary systems, except that of GRI–PVP K12, whereby the melting point depression method was used.

b PEtOx = poly­(2-ethyl-2-oxazoline); 5, 50, and 500 = PEtOx *M*_w_ of 5 000, 50 000, and 500 000 g mol^–1^, respectively.

## 3 Results and Discussion

### 3.1 Physicochemical Properties of Pure Components

The PC-SAFT modeling framework applied in this study relies on the key physicochemical properties of both the API and polymer components. For the APIs, *T*_m, onset_, Δ_fus_ *H*, and Δ_fus_ *C*_*p*_ were required. These values were obtained from previously published sources and are summarized in Table 3. For the polymers, the only required property was *M*_w_, which was previously reported in Table 1.

**Table 3:** Physicochemical Properties of the APIs Considered in This Work, Which Were Used in the PC-SAFT Calculations

**Compound**	**Form (CSD refcode)** ^d^	***T***_**m, onset**_ ^e^/ °C	**Δ_fus_** ***H*** ^f^/ kJ mol^–1^	**Δ_fus_** ***C*_*p*_** / J mol^–1^ K^–1^
FEL^a^	I (DONTIJ)	141.7	30.4 ± 1.0	89.9
GRI^b^	I (GRISFL25)	218.7	37.9 ± 1.1	93.8
IBU^a^	I (IBPRAC)	75.8	26.4 ± 0.8	176.2 – 0.3·*T*
IND^c^	γ (INDMET)	160.2	38.1 ± 1.1	238.2 + 0.3·*T*
NAP^b^	I (COYRUD11)	156.0	32.4 ± 1.0	99.3
NIF^b^	α (BICCIZ)	172.6	39.3 ± 1.2	121.22
PCM^a^	I (HXACAN34)	168.2	27.1 ± 0.8	99.8
PZQ^a^	A (TELCEU)	135.6	23.6 ± 0.7	103.3

a Taken from Mathers et al.^18^

b Taken from Mathers et al.^17^

c Taken from Mathers et al.^15^

d The exact polymorphic form considered.

e The uncertainty in the measured value is (*T* ± 0.3) °C.

f The uncertainty in the measured value is 3%.

### 3.2 Fitting of PC-SAFT Parameters to Experimental Solubility Data Using a Custom Optimization Program

PC-SAFT parameters were fitted to previously published experimental solubility data using the two-step optimization framework described in Section 2.3. The analysis in this section focused on polymeric excipients for which literature parameters are already available, namely, PVP K12, PVP VA 64, and SOL. These systems provide a benchmark to compare fitted versus literature parameters in downstream performance analyses. The extension of this methodology to PEtOx—a polymer with no reported PC-SAFT parameters—is presented separately in Section 3.5.

Table S3a and Table S3b in the Supporting Information list the optimized PC-SAFT parameters obtained during the first and second steps, respectively, for each API–polymer system. In all cases, the parameters were regressed directly to experimental API solubility (weight fraction) values without using a binary interaction parameter (i.e., *k*_ij_ = 0). As detailed later in Sections 3.3 and 3.4, these fitted values were used to evaluate the transferability of polymer parameters across APIs and the consistency of API parameters across different polymers.

### 3.3 Influence of Polymer Parametrization Strategy: PVP K12 as a Case Study

A key challenge in applying PC-SAFT to API–polymer systems is the selection of polymer parameters, especially in cases in which no experimental or literature data are available. This section focuses on PVP K12—a widely studied carrier polymer—to assess how different polymer parametrization strategies impact solubility modeling accuracy when the API parameters are held constant. By isolation of the polymer contribution, the comparative performance and transferability of alternative parametrization strategies can be evaluated directly. Three approaches were considered. In Approach 1, PC-SAFT parameters for both API and PVP K12 were taken directly from the literature. In Approach 2, the API parameters were fixed to their literature values, while the PVP K12 parameters were derived by fitting to the felodipine (FEL)–PVP K12 system using the custom optimization program developed in this work. In Approach 3, the API parameters again remained fixed, but the polymer parameters were taken as the average of those obtained—using the same fitting program—across all eight API–PVP K12 systems. Thus, both Approaches 2 and 3 relied on polymer parameters generated through data-driven regression to experimental solubility data rather than literature sources.

As shown in Figure 1, the untuned results (with *k*_ij_ = 0) reveal notable differences among the three strategies. The literature-based polymer values (LIT_0) offered the most reliable baseline performance, with an average RMSRD of 19.83. The FEL-based polymer parameters (FEL_0), although fitted specifically to one system, performed poorly when applied to other APIs, suggesting limited transferability of single-system-fitted polymer values. The averaged PVP K12 parameters (AVG_0) yielded better untuned accuracy than FEL0 in several systems (e.g., IND, NIF, PCM), indicating some benefit in generalizing across multiple fitted data sets. Upon optimization of the binary interaction parameter *k*_ij_, all three strategies converged toward comparably accurate fits. The average RMSRD values dropped to 2.92 (LIT_OPT), 2.91 (FEL_OPT), and 3.52 (AVG_OPT), with all systems achieving values below 5. Notably, even the initially poor-performing FEL-based parameters could yield high-quality fits following modest tuning, reinforcing the compensatory power of the *k*_ij_ term. The averaged polymer parameters consistently outperformed FEL-based values in the untuned state, making them a practical choice when literature data are unavailable and system-specific fitting is infeasible.

**Figure 1:**
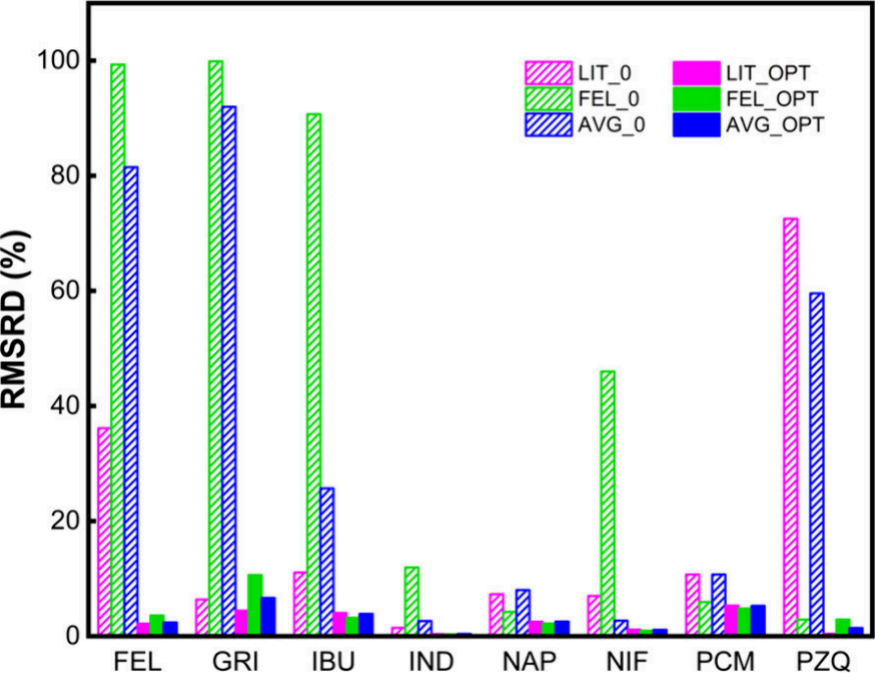
RMSRD values for the eight API–PVP K12 systems using three polymer parametrization strategies, shown before (0) and after (OPT) *k*_ij_ optimization. LIT = literature polymer values; FEL = felodipine-based fitted polymer values; AVG = average fitted polymer values.

Overall, these results highlight that while the selection of polymer parameters significantly affects untuned predictive performance, their influence diminishes once *k*_ij_ is adjusted. Polymer parameters derived through targeted regression to API–polymer solubility data—whether system-specific or averaged—can serve as viable surrogates when experimental or literature-based values are unavailable, provided that interaction tuning is permitted.

### 3.4 API Parametrization across Different Polymers: Consistency and Transferability

To explore the robustness and transferability of API-specific PC-SAFT parameters, naproxen (NAP) and nifedipine (NIF) were selected for comparative fitting across three chemically distinct polymers: PVP K12, PVP VA 64, and Soluplus (SOL). In this analysis, the PC-SAFT parameters for each polymer were held constant (as fitted in Section 3.2), while the five API parameters were reoptimized to each experimental solubility data set independently. This approach allowed assessment of whether a consistent parameter set for an API could reasonably describe its behavior across diverse polymer matrices. The derived API parameters showed moderate variability across the polymers (Table S3a and Table S3b in the Supporting Information), particularly in the dispersion energy term *u*/*k*_B_ and the hydrogen bonding parameters. For NAP, the *m*/*M*_w_ and σ values remained relatively stable, but *ε* ^assoc^/*k*_B_ ranged from ∼500 K (with SOL) to >2100 K (with PVP VA 64). Similarly, NIF showed changes in both σ and *u*/*k*_B_ depending on the polymer, while *ε* ^assoc^/*k*_B_ remained above 1000 K in all cases. These variations suggest that fitted API parameters are not strictly transferable across polymeric environments, likely reflecting real changes in API–polymer interactions (e.g., polarity and sterics) that influence solubility behavior.

The performance of these parametrizations is reflected in the RMSRD values (Figure 2). When the API parameters were taken from their fit to the PVP K12 system and transferred to PVP VA 64 or SOL, the untuned RMSRD values (*k*_ij_ = 0; bars labeled PVP K12_0) were generally low for NAP–PVP VA 64 and NIF–PVP VA 64—indicating reasonable baseline compatibility. However, the same transferred parameters performed poorly for SOL systems with RMSRD values above 75 for NAP and nearly 30 for NIF in the absence of tuning. This reflects the greater structural and chemical difference of SOL compared to those of the two PVP-based polymers. After *k*_ij_ optimization (bars labeled PVP K12_OPT), all systems achieved low RMSRD values (typically <3), demonstrating once again that minor interaction tuning can effectively compensate for mismatches in parameter transfer. Notably, the average-based parameter approach (AVG_0 and AVG_OPT) also showed a trend similar to the PVP K12-derived values: acceptable baseline fits for PVP VA 64 but poorer performance with SOL unless tuned. These observations reinforce that PC-SAFT API parameters fitted to one polymer should not be assumed transferable to structurally distinct carriers without additional refinement.

**Figure 2:**
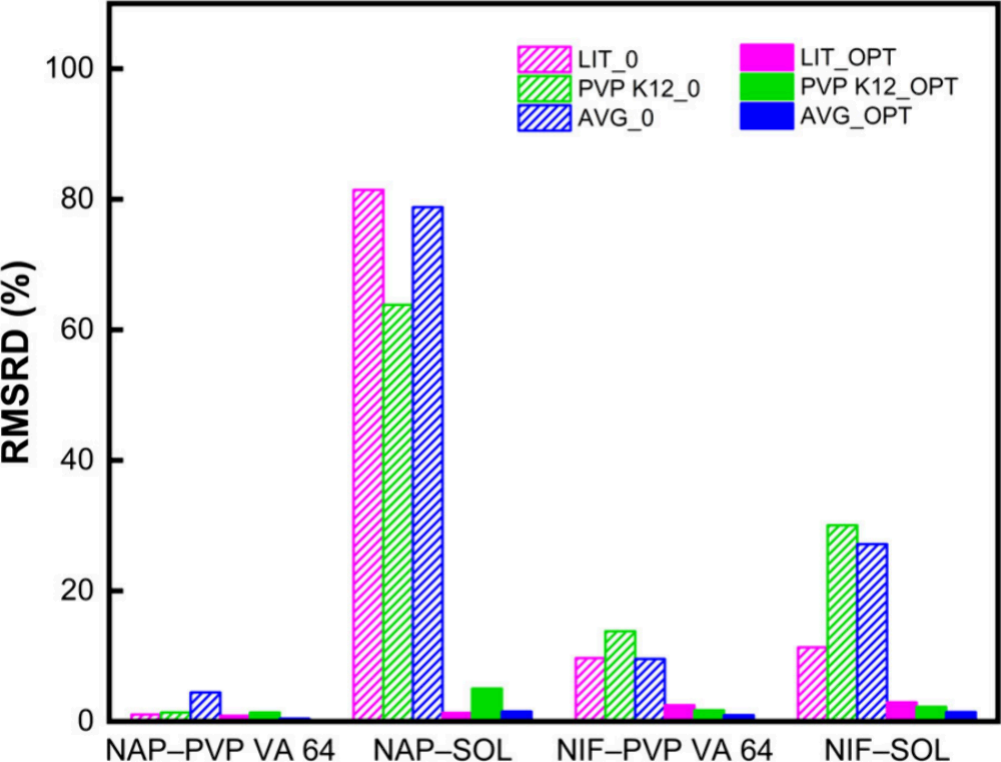
RMSRD values for the two API–PVP VA 64 and two API–SOL systems using three API parametrization strategies, shown before (0) and after (OPT) *k*_ij_ optimization. LIT = literature API values; PVP K12 = PVP K12-based fitted API values; AVG = average fitted API values. The optimized *k*_ij_ values for each binary system is provided in Table S2 in the Supporting Information.

Overall, this section highlights that API-specific PC-SAFT parameters are sensitive to the polymer matrix used during fitting. While values obtained from similar excipients (e.g., PVP K12 and PVP VA 64) can offer reasonable approximations, broader transferability—especially to chemically different polymers—requires caution. Tuning of *k*_ij_ remains essential when extrapolating between such systems.

### 3.5 Parametrization of API–PEtOx Systems: Establishing Empirical Starting Values

Unlike the polymers discussed in previous sections—namely, PVP K12, PVP VA 64, and SOL—poly­(2-ethyl-2-oxazoline) (PEtOx) lacks any reported PC-SAFT parameters in the literature. To address this gap, the two-step optimization workflow described in Section 2.3 was applied to generate empirically fitted PC-SAFT parameters for 12 binary API–polymer systems. These systems combined four APIs (IBU, IND, NAP, and PCM) with three PEtOx grades of varying molecular weight: 5,000 (PEtOx-5), 50,000 (PEtOx-50), and 500,000 (PEtOx-500) g mol^–1^. Regarding the value of *N* ^assoc^ for each PEtOx grade, the strategy that was adopted for PVP in Prudic et al.^5^ was followed in this study.

The optimized PC-SAFT parameters obtained from Step 1 and Step 2 fitting are provided in Table S4a and Table S4b, respectively. After extended optimization (Step 2), most systems achieved low RMSRD values, typically below 2.0, with notable examples including the IBU–PEtOx-5 and IND–PEtOx-5 systems, which yielded RMSRDs of 0.79 and 0.74, respectively. It is important to note, however, that the goal of this section is not to produce fully rigorous or universally transferable parameters. Rather, these values serve as reasonable starting points for systems lacking pure-component thermodynamic data. As observed for PVP K12, PVP VA 64, and SOL, tuning of the binary interaction parameter *k*_ij_ remains essential to achieving acceptable solubility predictions. Accordingly, using these parameters without *k*_ij_ optimization is not advised.

Table 4 presents the average PC-SAFT parameters for each PEtOx grade, intended to provide initial reference values for this polymer for which no prior parameters have been reported. The average parameters remain within the predefined fitting bounds and exhibit limited variation across the four API–PEtOx systems per grade, demonstrating internal consistency. A slight increase in dispersion energy (*u*/*k*_B_) with molecular weight is observed, but no systematic trends are evident across the full parameter set. This indicates that the chain length does not solely govern parameter behavior and underscores the complexity of API–polymer interactions. Nonetheless, these parameters should be regarded as preliminary and are expected to require further tuning—particularly of the binary interaction parameter—to enable accurate solubility predictions.

**Table 4:** Average PC-SAFT Parameters for PEtOx Grades Based on Simultaneous Fitting to Experimental DSC-Based Solubility Data of Four API–PEtOx Binary Systems

**Polymer**	***m***/***M***_w_ / mol g^–1^	**σ** / Å	***u***/***k***_B_ / K	***ε*** ^assoc^/***k***_B_ / K	**κ** ^assoc^
PEtOx-5	0.044196	2.880186	239.926701	0	0.02
PEtOx-50	0.048508	2.823947	240.522609	0	0.02
PEtOx-500	0.041947	2.855100	255.331741	0	0.02

This analysis further supports the versatility of the PC-SAFT framework in modeling API–polymer systems, even when literature parameters are absent. Additionally, the use of the mass-normalized segment number (*m*/*M*_w_) allows consistency in parameter scaling across polymers of different chain lengths, offering a practical and generalizable approach for ASD systems involving chemically novel polymers like PEtOx. The next section builds on these findings by questioning whether any carefully fitted parameters are necessary at all, particularly when comparable results can be obtained from arbitrary inputs once *k*_ij_ is optimized.

### 3.6 Literature Vs Arbitrary Parameters: Is Detailed Parametrization Necessary?

To evaluate the necessity of compound-specific PC-SAFT parametrization in API–polymer modeling, a final comparison was made using a single, fixed set of arbitrary parameter values applied uniformly across all API–PVP K12 systems. These values were selected as the midpoints of the literature-derived ranges and were not tailored to any particular compound. As shown in Figure 3, the arbitrary parameters produced poor baseline predictions when *k*_ij_ = 0 (ARB_0), with RMSRD values exceeding those from the literature-based sets (LIT_0) in nearly all cases. However, once the binary interaction parameter *k*_ij_ was optimized (ARB_OPT), the fit improved drastically. The final RMSRD values closely matched those achieved using optimized literature parameters (LIT_OPT), and in several systems (e.g., GRI, PCM), ARB_OPT yielded slightly better agreement with the experimental data. This suggests that detailed compound-specific parametrization may not be strictly necessary for achieving accurate solubility predictions, provided that *k*_ij_ is adjusted accordingly.

**Figure 3:**
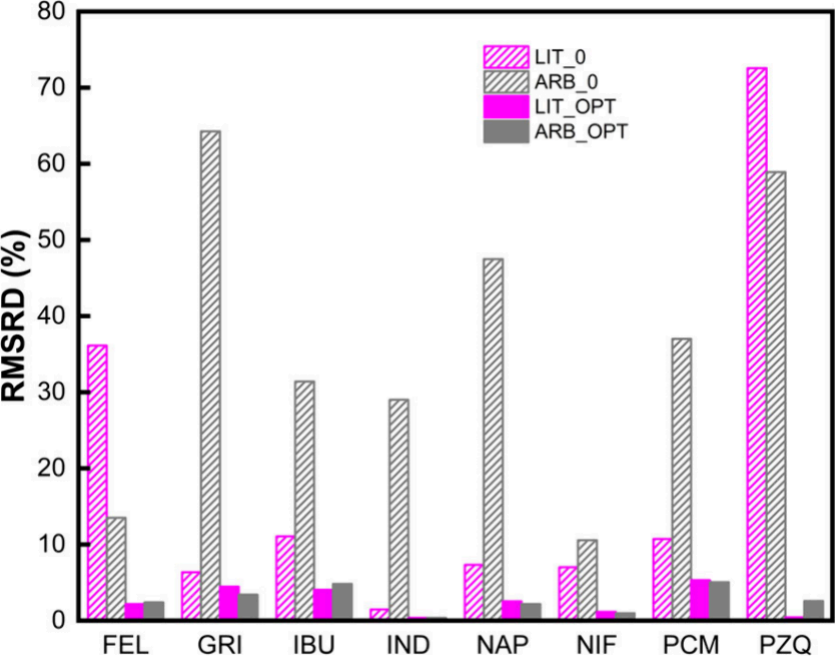
RMSRD values for the eight API–PVP K12 systems using two parametrization strategies, shown before (0) and after (OPT) *k*_ij_ optimization. LIT = literature API and PVP K12 values; ARB = arbitrary API and polymer values. The optimized *k*_ij_ values for each binary system is provided in Table S2 in the Supporting Information.

Figure 4 illustrates this behavior more clearly for the NAP–PVP K12 system. While LIT_0 gives a markedly better prediction than ARB_0, both SLE curves converge after *k*_ij_ optimization with LIT_OPT and ARB_OPT nearly overlapping across the entire temperature range. This example reinforces the broader conclusion: although literature-based parameters may, in general, yield better initial agreement, the final model accuracy is largely governed by the binary interaction parameter. Thus, arbitrary parameters—if chosen within reasonable bounds—can serve as practical stand-ins when no literature values are available, so long as *k*_ij_ is appropriately fitted.

**Figure 4:**
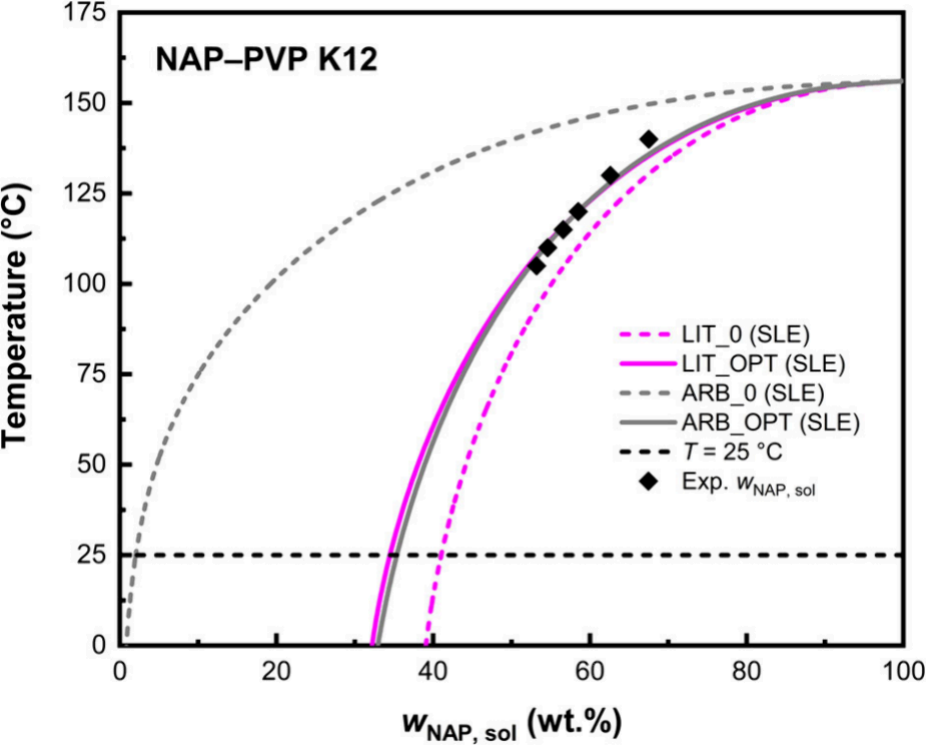
A comparison between the literature- and arbitrary-based SLE curves modeled by using PC-SAFT.

These findings highlight a key insight: with appropriate tuning of *k*_ij_, PC-SAFT predictions can be rendered highly accurate even when starting from approximate or nonspecific parameter sets. This observation challenges the prevailing assumption that rigorous fitting to pure-component thermophysical data (e.g., vapor pressure and liquid density) is essential for effective solubility modeling in amorphous systems. In practice, such data are often unavailable for new chemical entities, and their predictive value for API–polymer compatibility is limited—especially when interaction parameters must be tuned regardless. Thus, for API–polymer SLE modeling, the combination of flexible *k*_ij_ optimization and basic empirical fitting to solubility data may offer a more efficient and equally accurate pathway than traditional parametrization routes. This further supports the positioning of PC-SAFT as a pragmatic extrapolation framework rather than a purely predictive model based on fundamental molecular properties.

## 4 Conclusion

This work demonstrates the effective use of the PC-SAFT equation of state as a framework for extrapolating experimental drug–polymer solubility data, offering a practical strategy for extending known data into unmeasured conditions. By directly fitting PC-SAFT parameters to experimental solubility values and subsequently tuning the binary interaction parameter *k*_ij_, accurate modeling was achieved across a wide range of polymer systems, including both established (i.e., PVP K12, PVP VA 64, Soluplus) and less-characterized (i.e., poly­(2-ethyl-2-oxazoline)) materials. A key finding was that, for extrapolative purposes, the choice of initial pure-component parameters—whether drawn from literature, empirically derived from a single system, averaged across systems, or even selected arbitrarily within reasonable bounds—had minimal impact on final model accuracy once *k*_ij_ was optimized. This suggests that within this context PC-SAFT can serve as a robust and flexible solubility modeling tool even in the absence of exhaustive thermophysical characterization. However, these results also underscore the limitations of current parametrization strategies for pure prediction, where *k*_ij_ = 0. In such cases, differences in parameter selection did meaningfully affect the model performance. This highlights a continuing need for methodologies that yield physically meaningful parameters capable of generalizing across systems without requiring empirical adjustment. As such, while the present study supports a practical, data-fitted approach to PC-SAFT parametrization for formulation modeling, it also reinforces the value of ongoing efforts to improve predictive transferability for broader applications.

## Supplementary Material



## Notes

PC-SAFT parameter sets found in the literature, and the subsequently determined range values; PC-SAFT parameter sets obtained for the drug–polymer binary systems considered in the work using the proposed optimization framework (PDF)
