# Supplementary material for: A Parameter-Fitted PC-SAFT Framework for Solubility Extrapolation in Drug–Polymer Systems
Source: Mol Pharm. 2025 Sep 26;22(11):6956–63. doi: 10.1021/acs.molpharmaceut.5c00939 (PMC12587440; doi:10.1021/acs.molpharmaceut.5c00939)
Supplement: Supplementary file 1 [file mp5c00939_si_001.pdf]

A parameter-fitted PC-SAFT framework for solubility extrapolation in drug–polymer systems

Supporting Information

Alex Mathers [1], Michal Fulem [1,*]

[1] *Department of Physical Chemistry, University of Chemistry and Technology, Prague, Technická 5, 166 28 Prague 6, Czech Republic*

*Corresponding authors: fulemm@vscht.cz

Table S1a. PC-SAFT parameters for drugs reported in the literature (ref. = reference).

Compound	m/M_w	σ (Å)	u/k_B (K)	ε^{assoc}/k_B (K)	κ^{assoc}	ref.
artemisinin	0.02424	2.9590	293.7630	0.0	0.02	[1]
carbamazepine	0.04223	2.6580	151.6000	1094.1000	0.02	[2]
cinnarizine	0.03680	3.0860	231.0000	983.4000	0.02	[2]
felodipine	0.03000	3.2050	234.5000	1581.1000	0.02	[2]
fenofibrate	0.01070	4.7670	244.8000	0.0	0.02	[2]
glibenclamide	0.03700	3.0580	221.1000	2181.9000	0.02	[2]
griseofulvin	0.04018	3.3720	221.3000	1985.5000	0.02	[2]
ibuprofen	0.01223	4.4320	374.7000	879.4000	0.03	[2]
ibuprofen	0.02637	4.0179	309.4000	516.4691	0.089457	[3]
indomethacin	0.02207	3.8225	374.5100	1295.4320	0.011350	[3]
indomethacin	0.03992	3.5350	262.8000	886.4000	0.02	[2]
naproxen	0.01916	4.1142	470.9200	1202.6520	0.009524	[3]
naproxen	0.03522	2.9390	229.5000	934.2000	0.02	[2]
nifedipine	0.02347	3.5810	309.4400	1221.5800	0.02	[4]
paracetamol	0.02141	3.9819	432.0900	1635.9150	0.054320	[3]
paracetamol	0.04980	3.5080	398.2840	1994.2000	0.01	[5]
praziquantel	0.01991	4.0900	327.1000	0.0	0.02	[2]
probucol	0.02293	3.8500	175.6200	1650.0000	0.01	[6]
ritonavir	0.02200	3.9000	305.7870	1040.9700	0.02	[7]
simvastatin	0.01070	3.0711	296.0700	1449.5800	0.01	[8]
simvastatin	0.02480	3.7780	255.2300	1843.1000	0.02	[9]
Minimum value	0.01070	2.65800	151.60000	516.46910	0.00952	
Maximum value	0.04980	4.76700	470.92000	2181.90000	0.08946	

Table S1b. PC-SAFT parameters for polymers reported in the literature.

Compound	m_i/M_w	σ (Å)	u/k_B (K)	ε^{assoc}/k_B (K)	κ^{assoc}	ref.
HPC-SSL	0.04467	2.9740	205.00	1204.9	0.02	[10]
HPC-UL	0.04467	2.9740	205.00	1204.9	0.02	[10]
HPMC HME 100LV	0.04810	2.8890	298.05	1602.3	0.02	[11]
HPMCAS 716G	0.04366	2.8890	298.05	1602.3	0.02	[11]
PDLA	0.03699	3.1200	240.00	0.0	0.00	[12]
PGA	0.03130	2.8600	233.90	0.0	0.00	[12]
PLLA	0.04545	2.9200	230.00	0.0	0.00	[12]
PVP K12	0.04070	2.7100	205.599	0.0	0.02	[13]
PVP K25	0.04070	2.7100	205.599	0.0	0.02	[13]
PVP K30	0.04070	2.7100	205.599	0.0	0.02	[13]
PVP VA 64	0.03720	2.9470	205.271	0.0	0.02	[13]
Soluplus®	0.05400	2.8090	225.000	0.0	0.02	[14]
Minimum value	0.03130	2.71000	205.00000	– [a]	– [a]	
Maximum value	0.05400	3.12000	298.04700	– [a]	– [a]	

[a] For this particular study, the ε^{assoc}/k_B and k^{assoc} values for the polymer were set to 0.0 and 0.02, respectively.

Table S2. The optimized k_{ij} values calculated for each of the API–polymer binary systems in Figure 2 and Figure 3 from the main scientific article.

**API–polymer**	**Basis**	**Optimized k_{ij}**
NAP–PVP VA 64	LIT	0.001
	PVP K12	0.001
	AVG	0.011
NAP–SOL	LIT	0.059
	PVP K12	0.100
	AVG	0.071
NIF–PVP VA 64	LIT	–0.007
	PVP K12	0.016
	AVG	0.011
NIF–SOL	LIT	0.008
	PVP K12	0.036
	AVG	0.033
FEL–PVP K12	LIT	–0.032
	ARB	–0.013
GRI–PVP K12	LIT	–0.001
	ARB	0.015
IBU–PVP K12	LIT	–0.037
	ARB	–0.039
IND–PVP K12	LIT	–0.004
	ARB	–0.042
NAP–PVP K12	LIT	0.022
	ARB	–0.060
NIF–PVP K12	LIT	0.008
	ARB	–0.007
PCM–PVP K12	LIT	0.019
	ARB	–0.036
PZQ–PVP K12	LIT	0.064
	ARB	0.018

Table S3a. The optimized PC-SAFT parameters obtained after the first step for each API–polymer experimental $w_{API, sol}$ dataset.

API–polymer	m/M_w / mol g^{-1}	σ / Å	u/k_B / K	ε^{assoc}/k_B / K	κ^{assoc}	RMSRD
API–PVP K12						
FEL	0.012927	3.776194	368.468385	1921.818254	0.051395	1.6778
PVP K12	0.044467	2.722278	285.020716	0	0.02	
GRI	0.024723	3.117709	214.706743	1200.090701	0.020511	4.6181
PVP K12	0.039945	2.911826	216.226112	0	0.02	
IBU	0.023646	4.589366	450.141528	735.371990	0.017690	3.8989
PVP K12	0.032951	2.830620	251.516041	0	0.02	
IND	0.040468	4.145493	403.270029	986.942978	0.053143	2.5552
PVP K12	0.050447	2.890997	266.778668	0	0.02	
NAP	0.017401	4.524922	409.305940	1725.628592	0.018503	3.1283
PVP K12	0.049565	3.037177	264.593978	0	0.02	
NIF	0.022795	3.908379	419.520316	1101.841520	0.054321	1.6648
PVP K12	0.031944	3.098935	231.839698	0	0.02	
PCM	0.038481	4.428099	413.342285	816.201427	0.078800	3.4486
PVP K12	0.039933	2.762217	246.735534	0	0.02	
PZQ	0.015840	2.846728	185.098359	1345.953730	0.064410	3.5499
PVP K12	0.046969	2.951378	246.004065	0	0.02	
API–PVP VA 64						
NAP	0.017573	4.192902	447.678223	2133.510730	0.036957	0.4086
PVP VA 64	0.034265	2.807691	231.713113	0	0.02	
NIF	0.043655	3.730121	432.887464	1035.318595	0.035278	4.5694
PVP VA 64	0.033154	2.824428	235.143795	0	0.02	
API–SOL						
NAP	0.019872	3.537338	376.671298	519.145588	0.033375	2.5328
SOL	0.038893	2.818342	210.705912	0	0.02	
NIF	0.026948	4.415226	323.298380	1545.101880	0.027971	1.4714
SOL	0.038466	2.961039	237.924576	0	0.02	

Table S3b. The optimized PC-SAFT parameters obtained after the second step for each API–polymer experimental $w_{\mathrm{API,\ sol}}$ dataset.

**API–polymer**	**m/M_w / mol g^{-1}**	**σ / Å**	**u/k_B / K**	**ε^{assoc}/k_B / K**	**κ^{assoc}**	**RMSRD**
API–PVP K12						
FEL	0.013593	3.846281	367.984288	1942.521322	0.051120	1.3867
PVP K12	0.043720	2.716281	285.008733	0	0.02	
GRI	0.025915	3.073953	221.527844	1242.246763	0.021714	3.4103
PVP K12	0.035757	2.870823	212.148090	0	0.02	
IBU	0.023657	4.743811	486.342815	798.747513	0.019685	3.1474
PVP K12	0.038413	2.132834	219.168903	0	0.02	
IND	0.037549	3.876078	417.084823	1023.891198	0.055140	0.3222
PVP K12	0.052016	2.855400	272.279934	0	0.02	
NAP	0.015743	5.832508	407.300914	1498.894244	0.017703	0.6550
PVP K12	0.055580	2.784876	248.657032	0	0.02	
NIF	0.025442	3.965992	373.846792	1080.923234	0.058247	0.6871
PVP K12	0.031111	3.089776	230.635401	0	0.02	
PCM	0.032339	4.628992	414.689901	786.495892	0.082345	3.3338
PVP K12	0.041063	2.551341	242.603661	0	0.02	
PZQ	0.017015	2.852456	187.412793	1336.445402	0.059845	3.2300
PVP K12	0.047829	2.951321	246.479286	0	0.02	
API–PVP VA 64						
NAP	0.018459	4.194631	448.169849	2120.691721	0.037270	0.3593
PVP VA 64	0.034194	2.807905	231.780148	0	0.02	
NIF	0.044066	3.655895	436.865629	1052.100776	0.035742	0.3258
PVP VA 64	0.033302	2.855268	237.273158	0	0.02	
API–SOL						
NAP	0.016209	3.529711	437.484046	572.898108	0.033405	1.0431
SOL	0.039277	2.798100	210.462426	0	0.02	
NIF	0.027105	4.168325	324.828834	1640.384965	0.029452	1.0210
SOL	0.038849	3.096156	239.497916	0	0.02	

Table S4a. The optimized PC-SAFT parameters obtained after the first step for each API–PEtOx experimental solubility dataset.

**API–polymer**	m/M_w / mol g^{-1}	**σ / Å**	u/k_B / K	ε^{assoc}/k_B / K	κ^{assoc}	**RMSRD**
IBU	0.043304	4.260582	348.829453	886.246973	0.066279	1.1117
PEtOx-5	0.054126	2.804700	207.628405	0	0.02	
IBU	0.015571	3.698733	333.755307	1017.593242	0.060721	1.4114
PEtOx-50	0.052676	2.820700	210.469027	0	0.02	
IBU	0.026680	4.412275	387.972361	1602.190218	0.076136	3.7807
PEtOx-500	0.039058	3.033560	232.501559	0	0.02	
IND	0.038775	4.542545	460.701427	832.237544	0.059070	1.4287
PEtOx-5	0.049325	2.824644	255.073139	0	0.02	
IND	0.023941	4.223451	348.329573	1882.599240	0.080932	5.1218
PEtOx-50	0.050710	2.753530	234.567908	0	0.02	
IND	0.016273	4.473847	445.389800	1331.269654	0.083241	1.5375
PEtOx-500	0.048174	2.747858	274.690761	0	0.02	
NAP	0.040569	4.444919	459.578312	685.319747	0.010001	2.2057
PEtOx-5	0.031917	3.089884	283.426285	0	0.02	
NAP	0.029245	4.828732	369.810362	725.570054	0.050751	1.4055
PEtOx-50	0.049463	2.852088	250.534061	0	0.02	
NAP	0.032472	4.590384	427.419085	531.344596	0.011541	1.0244
PEtOx-500	0.035027	2.824507	256.029454	0	0.02	
PCM	0.032414	3.738548	315.719106	1204.906115	0.042938	9.3103
PEtOx-5	0.039860	2.909261	228.411441	0	0.02	
PCM	0.033625	4.286123	343.151735	653.250240	0.035862	2.0678
PEtOx-50	0.041183	2.869470	266.519441	0	0.02	
PCM	0.047229	4.024625	341.146653	1371.647636	0.035741	8.3580
PEtOx-500	0.046192	3.095953	251.116118	0	0.02	

Table S4b. The optimized PC-SAFT parameters obtained after the second step for each API–PEtOx experimental solubility dataset.

API–polymer	m/M_w / mol g^{-1}	σ / Å	u/k_B / K	ε^{assoc}/k_B / K	κ^{assoc}	RMSRD
IBU	0.043981	4.060867	354.279913	900.094582	0.067315	0.7941
PEtOx-5	0.054972	2.848523	205.033050	0	0.02	
IBU	0.016350	3.698733	333.755307	1017.593242	0.060721	1.1900
PEtOx-50	0.052676	2.820700	210.469027	0	0.02	
IBU	0.026680	4.412275	407.370979	1602.190218	0.076136	1.7584
PEtOx-500	0.039058	3.033560	232.501559	0	0.02	
IND	0.039260	4.599327	466.460195	842.640513	0.059808	0.7399
PEtOx-5	0.049942	2.859952	242.319482	0	0.02	
IND	0.023941	4.223451	365.746052	1882.599240	0.080932	1.9921
PEtOx-50	0.050710	2.753530	234.567908	0	0.02	
IND	0.016273	4.473847	445.389800	1397.833137	0.083241	1.5281
PEtOx-500	0.048174	2.747858	274.690761	0	0.02	
NAP	0.041319	4.843026	464.192521	706.064209	0.010143	1.8003
PEtOx-5	0.032484	3.038375	281.788928	0	0.02	
NAP	0.030707	4.828732	369.810362	725.570054	0.050751	1.0826
PEtOx-50	0.049463	2.852088	250.534061	0	0.02	
NAP	0.032472	4.590384	427.419085	557.911826	0.011541	1.0210
PEtOx-500	0.035027	2.824507	256.029454	0	0.02	
PCM	0.033762	4.360278	307.525864	1076.048350	0.044384	4.9045
PEtOx-5	0.039385	2.773895	230.565342	0	0.02	
PCM	0.033625	4.286123	343.151735	653.25024	0.035862	2.0679
PEtOx-50	0.041183	2.869470	266.519441	0	0.02	
PCM	0.048543	4.136638	337.148841	1409.823376	0.036736	6.4663
PEtOx-500	0.045529	2.814475	258.105190	0	0.02	

REFERENCES

(1) Prudic, A.; Ji, Y.; Sadowski, G. Thermodynamic Phase Behavior of API/Polymer Solid Dispersions. *Mol Pharm* **2014**, *11* (7), 2294-2304. DOI: 10.1021/mp400729x.

(2) Brinkmann, J.; Exner, L.; Verevkin, S. P.; Luebbert, C.; Sadowski, G. PC-SAFT Modeling of Phase Equilibria Relevant for Lipid-Based Drug Delivery Systems. *J Chem Eng Data* **2021**, *66* (3), 1280-1289. DOI: 10.1021/acs.jced.0c00912.

(3) Klajmon, M. Investigating Various Parametrization Strategies for Pharmaceuticals within the PC-SAFT Equation of State. *J Chem Eng Data* **2020**. DOI: 10.1021/acs.jced.0c00707.

(4) Luebbert, C.; Sadowski, G. In-situ determination of crystallization kinetics in ASDs via water sorption experiments. *Eur J Pharm Biopharm* **2018**, *127*, 183-193. DOI: https://doi.org/10.1016/j.ejpb.2018.02.028.

(5) Ruether, F.; Sadowski, G. Modeling the solubility of pharmaceuticals in pure solvents and solvent mixtures for drug process design. *J Pharm Sci* **2009**, *98* (11), 4205-4215. DOI: 10.1002/jps.21725.

(6) Zemánková, A.; Hassouna, F.; Klajmon, M.; Fulem, M. Solid-Liquid Equilibrium in Co-Amorphous Systems: Experiment and Prediction. *Molecules* **2023**, *28* (6). DOI: 10.3390/molecules28062492.

(7) Krummnow, A.; Danzer, A.; Voges, K.; Dohrn, S.; Kyeremateng, S. O.; Degenhardt, M.; Sadowski, G. Explaining the Release Mechanism of Ritonavir/PVPVA Amorphous Solid Dispersions. *Pharmaceutics* **2022**, *14* (9), 1904.

(8) Pavliš, J.; Mathers, A.; Fulem, M.; Klajmon, M. Can Pure Predictions of Activity Coefficients from PC-SAFT Assist Drug–Polymer Compatibility Screening? *Mol Pharm* **2023**, *20* (8), 3960-3974. DOI: 10.1021/acs.molpharmaceut.3c00124.

(9) Luebbert, C.; Stoyanov, E. Tailored ASD destabilization - Balancing shelf life stability and dissolution performance with hydroxypropyl cellulose. *Int J Pharm X* **2023**, *5*, 100187. DOI: https://doi.org/10.1016/j.ijpx.2023.100187.

(10) Luebbert, C.; Stoyanov, E.; Sadowski, G. Phase behavior of ASDs based on hydroxypropyl cellulose. *Int J Pharm X* **2021**, *3*, 100070. DOI: https://doi.org/10.1016/j.ijpx.2020.100070.

(11) Iemtsev, A.; Hassouna, F.; Mathers, A.; Klajmon, M.; Dendisová, M.; Malinová, L.; Školáková, T.; Fulem, M. Physical stability of hydroxypropyl methylcellulose-based amorphous solid dispersions: Experimental and computational study. *Int J Pharm* **2020**, *589*, 119845. DOI: https://doi.org/10.1016/j.ijpharm.2020.119845.

(12) Iemtsev, A.; Klajmon, M.; Hassouna, F.; Fulem, M. Effect of Copolymer Properties on the Phase Behavior of Ibuprofen–PLA/PLGA Mixtures. *Pharmaceutics* **2023**, *15* (2), 645.

(13) Lehmkemper, K.; Kyeremateng, S. O.; Heinzerling, O.; Degenhardt, M.; Sadowski, G. Long-Term Physical Stability of PVP- and PVPVA-Amorphous Solid Dispersions. *Mol Pharm* **2017**, *14* (1), 157-171. DOI: 10.1021/acs.molpharmaceut.6b00763.

(14) Wolbert, F.; Fahrig, I.-K.; Gottschalk, T.; Luebbert, C.; Thommes, M.; Sadowski, G. Factors Influencing the Crystallization-Onset Time of Metastable ASDs. *Pharmaceutics* **2022**, *14* (2), 269.
